# *Pasteurella multocida* and *Mannheimia haemolytica* Vaccines for Bovine Respiratory Disease: A Comprehensive Review

**DOI:** 10.3390/ani16182911

**Published:** 2026-09-16

**Authors:** Minyi Zhang, Zhijun Chen, Guangfu Zhao, Falong Yang, Qibing Gu

**Affiliations:** College of Animal & Veterinary Sciences, Southwest Minzu University, Chengdu 610041, China; 15892460650@163.com (M.Z.); czj0312@outlook.com (Z.C.); zgf515005117@outlook.com (G.Z.); falong.yang@swun.edu.cn (F.Y.)

**Keywords:** *Mannheimia haemolytica*, *Pasteurella multocida*, bovine respiratory disease, vaccines, serotype diversity

## Abstract

Bovine Respiratory Disease (BRD) is a leading cause of economic losses in the global cattle industry, with *Mannheimia haemolytica* and *Pasteurella multocida* as the primary bacterial pathogens. This review provides a comprehensive overview of vaccine research progress against these two pathogens, covering inactivated vaccines, live attenuated vaccines, subunit vaccines, DNA vaccines, recombinant vector vaccines, and outer membrane vesicle vaccines. Their immune mechanisms, protective efficacy, adjuvant applications, and field performance are discussed. Key challenges include maternal antibody interference, the increasing prevalence of *M. haemolytica* serotype A6 that evades current commercial vaccines, and the serotype diversity of *P. multocida*. Future vaccine development should focus on overcoming maternal antibody interference, enhancing cross-serotype protection, optimizing mucosal adjuvants and delivery systems, and developing multivalent vaccines covering major epidemic serotypes. Integrating reverse vaccinology, multi-epitope vaccine design, and nanotechnology holds promise for safer, more effective, and broader-spectrum next-generation vaccines.

## Simple Summary

Bovine Respiratory Disease (BRD) causes major economic losses in the global cattle industry, primarily due to *Mannheimia haemolytica* and *Pasteurella multocida*, which are growing increasingly antibiotic-resistant. This review summarizes vaccines developed against these bacteria, ranging from traditional inactivated types to newer DNA and protein subunit vaccines. Key challenges include limited cross-strain protection and reduced efficacy in calves due to maternal antibodies. Future strategies should prioritize broader strain coverage, intranasal delivery to enhance local respiratory immunity, and multi-antigen formulations. These advances aim to reduce disease and antibiotic use, improve cattle health and productivity, and support sustainable livestock farming.

## 1. Introduction

Bovine Respiratory Disease (BRD) represents a multifaceted condition that substantially undermines the economic viability of the global cattle industry, incurring annual losses amounting to billions of dollars [1]. Studies have found that the incidence of BRD is influenced by genetic, environmental and economic factors; castrated bulls are more susceptible than heifers, and the heritability of resistance to BRD is estimated to be between 0.04 and 0.08 [2]. As the principal bacterial pathogen of BRD, *M. haemolytica* (this organism was previously classified as *Pasteurella haemolytica* prior to the taxonomic reclassification in 1999) is the major cause of severe pneumonia in BRD [3] and accounts for most disease-associated mortalities [4]. Conversely, *P. multocida* functions primarily as a secondary pathogen within BRD but exhibits a broader host range, being implicated in diverse animal diseases including bovine hemorrhagic septicemia, fowl cholera, and atrophic rhinitis in swine [5]. Studies have already indicated that there is a clear association between capsular types and these diseases (type A/F causes fowl cholera, type B/E causes bovine haemorrhagic septicaemia, and type D causes atrophic rhinitis in pigs) [5,6]. This broad pathogenic potential poses considerable risks to the livestock industry, thereby necessitating targeted immunological control strategies [7]. Both *M. haemolytica* and *P. multocida* typically colonize the mucosal surfaces of the upper respiratory tract in cattle, maintaining a commensal relationship under healthy conditions. However, stressors such as transportation, concurrent viral infections, and suboptimal environmental factors can compromise host immune defenses. Stress-induced immunosuppression—driven by transport, viral co-infections, and environmental factors—primes cattle for secondary bacterial infection and subsequent bronchopneumonia [8].

The escalating issue of antimicrobial resistance warrants significant attention. Surveillance data from both domestic and international sources reveal a progressive increase in resistance rates of *M. haemolytica* and *P. multocida* to commonly employed traditional antibiotics, including tetracyclines, penicillins, and macrolides [9]. Recent systematic reviews have confirmed that resistance to tetracyclines, macrolides, sulfonamides, and β-lactams remains prevalent across North America, Europe, and Asia, with resistance prevalence ranging from 0.8% to 100% depending on the antimicrobial class and region [10]. In Europe, the German national resistance monitoring program GERM-Vet (2009–2020) documented a slowly increasing prevalence of macrolide resistance among BRD-associated *M. haemolytica* isolates, with multidrug-resistance-mediating integrative and conjugative elements (ICEs) carrying resistance genes including *erm* (T), *mef* (C), *mph* (G), *floR*, *tet* (Y), and *sul2* [11]. A retrospective analysis of publicly available genomic data from North America further revealed that clinically relevant antimicrobial resistance genes (ARGs) are commonly encoded by *M. haemolytica* and *P. multocida*, with the tylosin esterase-encoding gene *estT* being among the most frequently observed ARGs in feedlot-associated metagenomic datasets [12]. In Asia, genomic surveillance has similarly identified a broad array of resistance genes in BRD pathogens, highlighting the global distribution of multidrug-resistant strains [10]. Multidrug-resistant strains have become prevalent, with certain isolates demonstrating diminished susceptibility even to novel antibiotics or drugs reserved for clinical use [9]. The detection of macrolide resistance genes alongside other resistance genes on MDR-mediating ICEs in bovine *M. haemolytica* may explain the occurrence of therapeutic failure when treating BRD with regularly used antimicrobial agents [11]. This trend compromises conventional antimicrobial therapy, leading to frequent treatment failures, disease recurrence, and increased risks of drug residues. In the context of global initiatives aimed at limiting the use of antibiotic growth promoters and reducing dependence on pharmacological interventions, exclusive reliance on antimicrobial therapy is increasingly unsustainable. Consequently, the advancement of safe and efficacious vaccines, alongside the establishment of prevention-focused disease control systems, has emerged as a critical strategy for managing these infections and promoting the sustainable development of the livestock sector [13].

It should be noted that bovine respiratory disease (BRD) is a polymicrobial complex involving multiple viral and bacterial pathogens. In addition to *M. haemolytica* and *P. multocida*, other bacterial agents such as *Histophilus somni* and *Mycoplasma bovis* are also recognized as important components of this disease complex. However, the present review deliberately focuses on *M. haemolytica* and *P. multocida* for the following reasons: (1) these two bacterial species are the most frequently isolated pathogens from BRD-affected cattle worldwide and are responsible for the majority of bacterial-associated mortality; (2) the vaccine research landscape for these two pathogens is substantially more extensive and advanced compared to that for *H. somni* and *M. bovis*, providing a richer body of literature for comprehensive analysis; and (3) they share similar pathogenic mechanisms, host colonization strategies, and vaccine development challenges (e.g., serotype diversity and the need for mucosal immunity), enabling a focused and coherent discussion. While *H. somni* and *M. bovis* are acknowledged as relevant contributors to BRD, they are beyond the scope of this review and are discussed only when directly relevant to comparative vaccine strategies or combination vaccine formulations. This review systematically examines the current landscape of vaccine research, immunological mechanisms, and developmental trajectories concerning *M. haemolytica* and *P. multocida*, with the objective of informing future research endeavors and guiding prevention and control measures.

## 2. Search Strategy

To comprehensively identify relevant literature, we searched PubMed, Web of Science, Scopus, and Google Scholar databases, covering publications from January 1990 to July 2026.

Search terms included: “Bovine Respiratory Disease”, “BRD”, “*Mannheimia haemolytica*”, “*Pasteurella multocida*”, “vaccine”, “vaccination”, “immunization”, “subunit vaccine”, “live attenuated vaccine”, “DNA vaccine”, “recombinant vaccine”, “outer membrane vesicle”, “OMV”, “vector vaccine”, “mucosal vaccine”, and “adjuvant”.

Inclusion criteria: (1) original research, clinical trials, or peer-reviewed reviews published in English; (2) studies on vaccines against *M. haemolytica* or *P. multocida*; (3) studies reporting immunogenicity, protective efficacy, or field performance; (4) studies addressing vaccine development challenges (e.g., maternal antibody interference, serotype diversity, adjuvants, delivery systems).

Exclusion criteria: (1) non-English publications; (2) conference abstracts, case reports, or opinion pieces without original data; (3) studies focusing exclusively on viral pathogens; (4) studies not reporting vaccine efficacy data; (5) publications before 1990 (except landmark studies).

The initial search yielded 847 records. After removing duplicates, 644 records remained. Following title and abstract screening, 178 full-text articles were assessed for eligibility, and 81 articles were ultimately included in this review.

## 3. Vaccines Against *M. haemolytica*

### 3.1. Inactivated Vaccines

At present, most commercially available vaccines targeting *M. haemolytica* are developed as inactivated formulations. These vaccines employ chemical or physical inactivation techniques, such as treatment with formaldehyde or heat, to neutralize bacterial virulence while maintaining immunogenic properties. Proteomic analyses have demonstrated that effective commercial vaccines incorporate several critical immunogenic components, including leukotoxin A (LktA), outer membrane proteins (e.g., Ssa1, OmpP6), iron-binding proteins (YfeA), and ABC transporters [14].

Advantages and limitations of inactivated vaccines: Inactivated vaccines are characterized by a high safety profile and an inability to revert to virulence. However, they generally confer only transient immunity against homologous strains and necessitate multiple doses for sustained protection. Proteomic analyses suggest that effective commercial vaccines should contain adequate levels of LktA, which is the primary factor responsible for the clinical manifestations and lung injury caused by this disease [14,15]. Nonetheless, the quantity of LktA present in many commercial formulations is often suboptimal, thereby constraining their protective effectiveness [16]. Furthermore, inactivated vaccines have inherent limitations in eliciting robust cellular and mucosal immune responses. Studies have shown that following subcutaneous vaccination of sheep with a gamma-irradiated inactivated vaccine, serum IgG (humoral immunity) is significantly elevated and IL-4 (a Th2-type cytokine) is increased, whereas IFN-γ (a Th1-type cytokine and a key marker of cellular immunity) shows no significant change [17].

To overcome the low LktA yield in standard bacterins, recent studies have optimized culture conditions or supplemented vaccines with recombinant proteins to enhance protective immunity without sacrificing safety [16]. Moreover, the selection of adjuvants plays a crucial role in modulating both the magnitude and durability of the vaccine-induced immune response; adjuvants such as Freund’s adjuvant, aluminum-based compounds, and novel water-soluble formulations have each demonstrated unique benefits in enhancing the protective efficacy of inactivated vaccines [18,19]. Notably, a study conducted by Confer et al. (2009) revealed that cholera toxin, when employed as a mucosal adjuvant, significantly improves antigen presentation of the chimeric PlpE-LKT protein and facilitates the establishment of immune memory following intranasal immunization in calves [20].

### 3.2. Leukocyte Toxin (LktA) Vaccine

Leukotoxin A (LktA) represents the principal virulence determinant of *M. haemolytica* and is classified within the RTX (Repeats-in-Toxin) toxin family [21]. Distinct from other virulence factors, LktA demonstrates both cell-type and species specificity, predominantly targeting immune cells in ruminant hosts. The pathogenic mechanism of LktA involves its binding to CD18 (β2-integrin) receptors on the leukocyte surface, resulting in pore formation within the cell membrane. This process leads to leukocyte lysis and concurrently triggers the release of inflammatory mediators, thereby contributing to pulmonary tissue damage. Consequently, LktA has been identified as a critical antigenic target in the development of vaccines against *Mannheimia* infections.

Recombinant LktA Vaccine: The full-length LktA protein exhibits low expression levels and limited biological stability, prompting researchers to develop truncated recombinant variants to address these limitations. Recent investigations have demonstrated that the C-terminal domain of LktA (cLktA) possesses favorable immunogenic properties and enhanced stability [22]. Experimental studies in mice (intramuscular immunization) and goats (intramuscular immunization) have shown that a single administration of cLktA elicits robust antibody responses with high titers, conferring protection rates ranging from 80% to 100% against homologous challenge [22]. The cLktA fragment contains highly conserved, immunodominant epitopes, while its hydrophilic residues maintain the protein’s structural integrity and stability [22]. The protective immunity induced by the LktA vaccine is primarily mediated by neutralizing antibodies, which inhibit the interaction between the toxin and host cell receptors, thereby mitigating pulmonary tissue damage.

Limitations of the Recombinant LktA Vaccine: Vaccines exclusively targeting LktA present significant limitations. Primarily, the genetic sequences of the *LktA* gene differ among various serotypes of *M. haemolytica*, which may result in insufficient cross-protection against heterologous serotypes. Additionally, LktA and lipopolysaccharide (LPS) act synergistically during infection, enhancing pathogenicity. Consequently, administration of recombinant LktA (rLktA) alone has proven to be possibly inadequate in fully preventing the development of clinical symptoms in bovine models. These observations suggest that an effective *Mannheimia* vaccine should incorporate LktA alongside other protective antigens, such as outer membrane proteins and iron-binding proteins, to elicit broader and more sustained immune protection.

### 3.3. Outer Membrane Protein Vaccines

Proteomic analyses have identified multiple candidate proteins suitable for subunit vaccine development. Among these, Lipoprotein E (PlpE) [23], a predominant outer membrane protein with a molecular weight of 45 kDa, is consistently expressed across all serotypes. Antibodies directed against PlpE substantially enhance complement-mediated bactericidal activity; notably, the removal of these antibodies from immune serum results in a marked reduction in the capacity to eliminate *Mannheimia*. Recombinant PlpE (rPlpE) exhibits strong immunogenic properties; administration of a 100 μg dose significantly improves resistance to virulent challenges in cattle, and its inclusion in commercial vaccines markedly increases protective efficacy (*p* < 0.05) [24]. These characteristics are consistent with those of other immunogenic outer membrane proteins, which are surface-exposed, serve as targets for the host immune response, and hold promise as vaccine candidates [25].

Serotype-specific antigen 1 (Ssa1) is an outer membrane protein that elicits a robust antibody response and represents the most immunoreactive antigen candidate among the A1, A2, and A6 serotypes of *M. haemolytica*. The ssaI gene on pSSA1 encodes Ssa1, a roughly 100 kDa protein. Surface expression of Ssa1 in *E. coli* enabled its use as an immunogen to produce rabbit antiserum, confirming its immunogenicity. Additionally, colony immunoblot analysis using convalescent calf serum from animals resistant to *M. haemolytica* A1 pneumonia provided indirect evidence that Ssa1 is immunogenic in calves as well [26]. Additional candidate proteins include Omp P6 (a member of the OmpA family, homologous to proteins found in *H. somni* and *P. multocida*), the iron-binding protein YfeA, and ABC transporters such as OppA and HisJ-like proteins, all of which contribute significantly to immune protection. However, *M. haemolytica* OmpA exhibits high genetic variability within its hypervariable regions across different host strains, driving host-adaptive evolution and hindering the development of cross-protective vaccines [25].

Outer membrane protein subunit vaccines exhibit certain limitations and challenges. Specifically, the target proteins are susceptible to degradation throughout the processes of preparation and purification, and their intrinsic immunogenicity tends to be relatively low, often necessitating multiple administrations to achieve a robust immune response. Additionally, these vaccines generally require the incorporation of appropriate adjuvants to attain optimal protective efficacy [4]. Empirical evidence indicates that the combination of recombinant PlpE protein with commercially available vaccines can substantially improve immunoprotective outcomes [24].

### 3.4. Novel Vaccine Delivery Systems

A plant-based oral vaccine comprising three antigens—fragments of LktA and PlpE, along with the cholera toxin B subunit (CTB)—was generated utilizing the Nicotiana benthamiana expression system [27]. Oral administration of this vaccine to mice elicited specific IgG antibodies targeting LktA and PlpE, while mucosal IgA antibodies were detected in both pulmonary tissue and fecal samples, demonstrating the induction of a mucosal immune response [27]. Additionally, the lyophilized plant-derived material exhibited stable storage at ambient temperature for up to one year [27]. Plant expression platforms present several advantages, including reduced production costs, scalability, and the elimination of contamination risks associated with animal pathogens [28], thereby offering a viable and accessible vaccine production strategy for resource-limited settings.

Bacillus spore-based vaccine: This approach employs Bacillus subtilis spores as both an adjuvant and a delivery system to adsorb a chimeric protein (MhCP) comprising the neutralizing epitopes of LktA and PlpE [29]. In mice, intranasal administration of this vaccine elicits a robust secretory IgA response in bronchoalveolar lavage fluid, saliva, and feces, surpassing the immunogenicity of free antigens and exhibiting enhanced bactericidal efficacy against intranasal *M. haemolytica* challenge [30]. The mucosal vaccine confers protection by restricting pathogen colonization and proliferation within the respiratory tract, thereby demonstrating broad potential for application [30]. Additionally, Bacillus spores exhibit remarkable environmental stability and can be stored at ambient temperature for prolonged durations, substantially reducing the logistical costs associated with cold-chain storage and transportation of vaccines [31].

Nanoparticle Delivery Systems: Chitosan nanoparticles have shown great potential as DNA vaccine carriers and adjuvants in the field of veterinary immunization. Chitosan possesses characteristics such as biodegradability, biocompatibility, and low toxicity. Furthermore, its mucoadhesive properties facilitate cellular uptake and protect nucleic acids from enzymatic degradation [32]. Studies targeting *P. multocida* have demonstrated that chitosan-DNA nanoparticle formulations can effectively induce humoral and cellular immune responses, as well as enhance mucosal immunity, thereby improving the immunogenicity and protective efficacy of DNA vaccines [32,33]. Given that *M. haemolytica* and *P. multocida* are both important Gram-negative respiratory pathogens, similar chitosan-based nanodelivery strategies hold broad application prospects for the development of effective vaccines against *M. haemolytica*. However, research specifically focused on chitosan nanoparticle vaccines against *M. haemolytica* remains limited. Challenges such as insufficient field trial data, formulation stability, and standardization of manufacturing processes remain key areas for future research.

### 3.5. Serotype Diversity, Cross-Protection Challenges, and Emerging Vaccine Strategies

*M. haemolytica* is categorized into multiple serotypes, with serotypes A1, A2, and A6 being the most widely distributed [34]. Notably, the incidence of serotype A6 has increased markedly in recent years, establishing it as a predominant pathogen affecting both cattle and sheep populations [34]. Considerable phenotypic and genotypic variability is observed among these serotypes. Presently available commercial vaccines predominantly target serotypes A1 and/or A2, and do not confer cross-protection against serotype A6. This discrepancy between the serotypes included in commercial vaccines and those prevalent in the field contributes to suboptimal vaccine performance.

To address these challenges, the formulation of a trivalent vaccine targeting the A1, A2, and A6 serotypes has emerged as a critical objective. Recent investigations have demonstrated that a trivalent vaccine comprising whole cells of serotypes A1, A2, and A6, in combination with recombinant LktA (rLktA) and recombinant SSA-1 (rSSA-1), adjuvanted with Montanide™ ISA 206 VG, achieves a seroconversion rate of 95.8% and confers complete (100%) protection upon challenge, thereby markedly surpassing the efficacy of existing commercial vaccines [16].

Beyond multivalent whole-cell formulations, multi-epitope vaccines designed via immunoinformatics have emerged as a promising strategy to address serotype diversity. This approach utilizes bioinformatics to identify and concatenate linear B- and T-cell epitopes from conserved antigens [35,36]. Compared to conventional whole-cell bacterins, this strategy circumvents serotype-specific limitations by targeting conserved regions, enables precise immune activation by excluding immunosuppressive components, and eliminates virulence reversion risks since no live pathogen material is required [35,36]. Research on multi-epitope vaccines targeting *M. haemolytica* can be pursued through several key avenues. Firstly, building upon prior investigations [16], it is essential to identify B-cell and T-cell epitopes that are conserved across different serotypes. Secondly, incorporating data on antigens that are either serotype-specific or shared within the A6 serotype can enhance the identification of optimal epitopes derived from this particular serotype. Lastly, the selected epitopes should be concatenated using flexible linkers to develop a multivalent recombinant antigen encompassing epitopes from serotypes A1, A2, and A6. Subsequently, the immunogenic potential of this multivalent antigen, when combined with novel adjuvants such as Montanide™ ISA 206 VG as demonstrated in previous trivalent vaccine formulations, warrants thorough evaluation.

**Figure 1:**
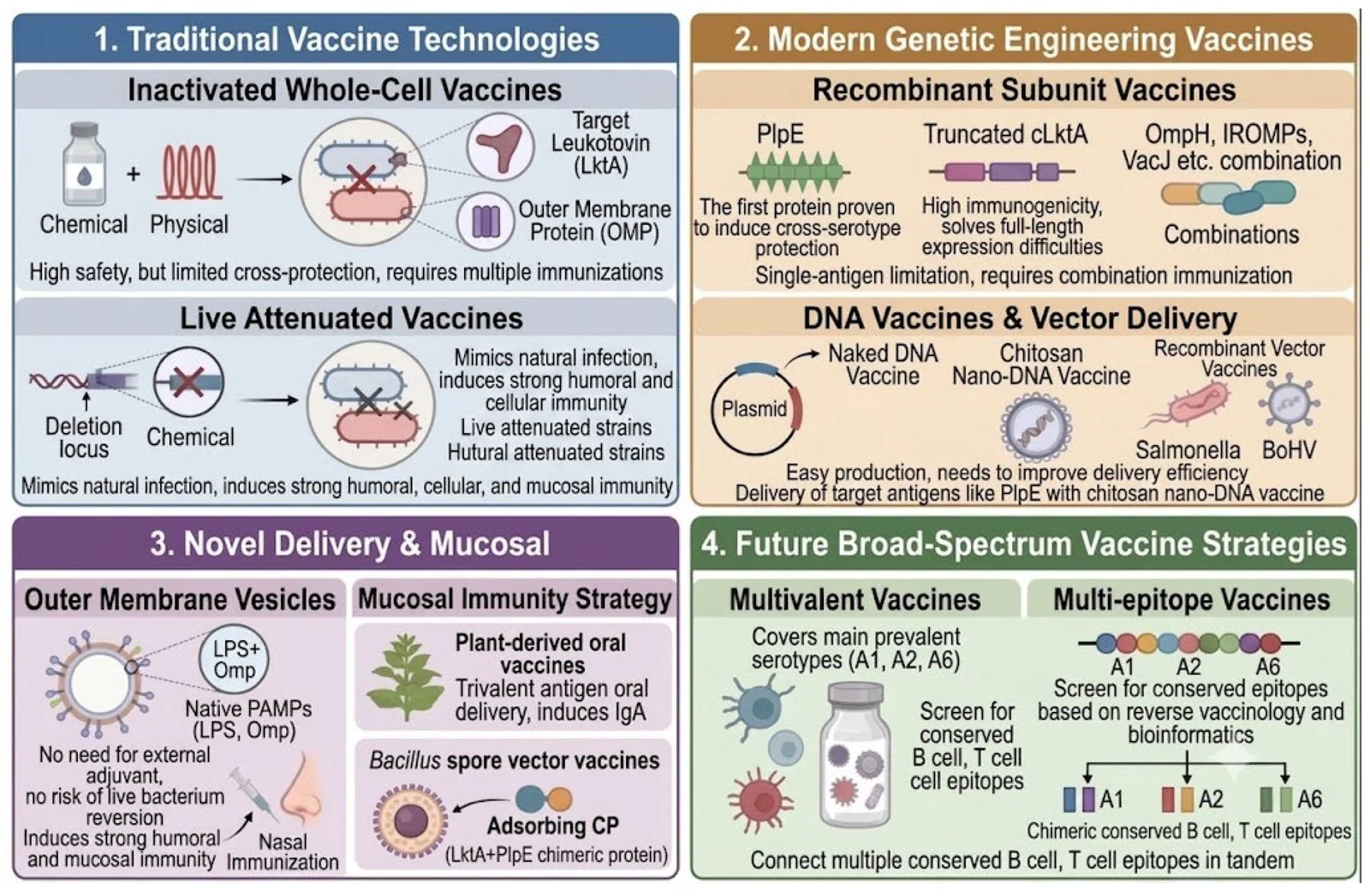
Vaccine development landscape for *M. haemolytica*. 1. Traditional Vaccine Technologies: Inactivated whole-cell vaccines (chemically or physically inactivated) offer high safety but limited cross-protection and require multiple immunizations; live attenuated vaccines mimic natural infection and induce strong humoral, cellular, and mucosal immunity but carry the risk of virulence reversion. 2. Modern Genetic Engineering Vaccines: Recombinant subunit vaccines (e.g., PlpE, truncated cLktA) exhibit high immunogenicity and solve full-length expression difficulties but face single-antigen limitations; DNA vaccines and vector-based delivery systems (e.g., chitosan nanoparticles, Salmonella, BoHV vectors) offer easy production but require improved delivery efficiency. 3. Novel Delivery & Mucosal Strategies: Outer membrane vesicle (OMV) vaccines contain natural PAMPs (pathogen-associated molecular patterns, e.g., LPS, OMPs), require no external adjuvant, carry no risk of live bacterium reversion, and induce strong humoral and mucosal immunity; Bacillus spore-based vaccines and intranasal immunization represent promising mucosal immunity strategies. 4. Future Broad-Spectrum Vaccine Strategies: Multivalent vaccines covering major prevalent serotypes (A1, A2, A6) and multi-epitope vaccines constructed by screening and linking multiple conserved B-cell and T-cell epitopes via bioinformatics represent the next-generation direction. Key targets include leukotoxin (LktA) and outer membrane proteins (OMPs).

In summary, the development of multivalent and multi-epitope vaccines represents a promising strategy to overcome challenges related to serotype variability and limited cross-protection in *M. haemolytica*. Future advancements are anticipated through the integration of immunoinformatics-driven epitope prediction, reverse vaccinology, high-throughput epitope screening techniques, and the utilization of nanodelivery systems. Such approaches are expected to facilitate the creation of novel genetically engineered *M. haemolytica* vaccines with enhanced breadth of coverage and improved protective efficacy (Figure 1). To facilitate systematic comparisons among different vaccine platforms, Table 1 summarizes the key characteristics of each *M. haemolytica* vaccine platform, including antigen composition, adjuvant/delivery system, immune response type, protective efficacy, advantages, limitations, and representative references.

## 4. Vaccines for *P. multocida*

### 4.1. Pathogenic Characteristics and Serotyping

*P. multocida* is classified into five capsular serogroups (A, B, D, E, and F) based on capsular polysaccharide antigens detected by indirect hemagglutination [37]. Additionally, strains can be typed according to the lipopolysaccharide (LPS) outer core biosynthesis locus, which has led to the identification of eight LPS genotypes (L1–L8) [38,39]. Capsular serogroups are closely associated with specific pathological conditions: types B:2 and E:2 are the primary causative agents of hemorrhagic septicemia in cattle and water buffalo; type A is highly correlated with fowl cholera, and certain type A strains are also associated with atrophic rhinitis in pigs and rabbits; toxigenic type D strains are more common in pigs and are often associated with disseminated infections [5]. The principal virulence determinants encompass capsular polysaccharides (CPS), lipopolysaccharides (LPS), outer membrane proteins (OMPs), iron-acquisition proteins such as transferrin-binding protein A (TbpA), and toxins including *P. multocida* toxin (PMT), all of which represent potential targets for vaccine development. Notably, *P. multocida*-derived outer membrane vesicles (OMVs) are rich in multiple pathogen-associated molecular patterns (PAMPs) and immunogenic proteins, including LPS, OmpA, OmpH, and BamA. They can be recognized and internalized by macrophages, thereby promoting phagocytic activity, cell proliferation, and cytokine secretion, which in turn activate innate and adaptive immune responses, demonstrating promising potential as subunit vaccine candidates [40].

### 4.2. Inactivated Vaccines

Currently, clinical vaccines against fowl cholera and hemorrhagic septicemia (HS) predominantly rely on formaldehyde-inactivated whole-cell formulations. In the control of bovine pasteurellosis, extensive research has focused on vaccine candidates targeting capsular serotypes A and B. However, the protective efficacy of these conventional inactivated vaccines varies considerably. As noted by Verma and Jaiswal in their review on HS vaccines [41], oil-adjuvanted inactivated vaccines (OAV) can provide higher levels and longer duration of immune protection (up to one year) compared to conventional bacterins, while the B:3,4 live vaccine developed in Myanmar confers immunity for over one year [41]. In recent years, next-generation HS vaccine candidates have emerged. A novel subunit vaccine targeting the surface lipoprotein PmSLP-3, formulated with Montanide ISA 61, has demonstrated sustained serum IgG titres for up to three years after two doses and full protection against both serogroup B and E challenge in cattle [42]. Outer membrane vesicle (OMV)-based vaccines derived from yak-origin *P. multocida* serogroup B have also shown promising immunogenicity, providing complete protection against homologous challenge following Intramuscular injection in mice [43]. Additionally, recombinant and DNA vaccine platforms continue to be explored as next-generation HS strategies [44]. Empirical investigations have indicated that inactivated vaccines targeting *P. multocida* exhibit limited efficacy in conferring cross-protection. Specifically, a study by Sayed et al. [45] revealed that while an inactivated vaccine derived from *P. multocida* serotype B achieved complete (100%) protection against homologous strains following Subcutaneous inoculation in mice, it failed entirely to protect against the heterologous serotype E, with all immunized mice succumbing to infection within 28 h following Subcutaneous inoculation challenge. Consistently, Guan et al. [46] reported that inactivated vaccines prepared from A:L3 genotype strains provided no cross-protection (0%) against heterologous A:L6 genotype strains in a murine intraperitoneal challenge model. These cumulative findings corroborate the long-standing observation that whole-cell bacterins elicit robust homologous immunity but demonstrate markedly reduced efficacy against heterologous variants, a historical limitation that continuous to challenge modern vaccine design.

It is widely acknowledged that although inactivated *P. multocida* vaccines provide strong protection against homologous strains, they are inherently limited by short-lived immunity and a narrow spectrum of cross-protection [47]. Therefore, enhancing the protective range of these vaccines and extending the longevity of the immune response—while maintaining their proven safety profile and effectiveness against homologous strains—constitutes a primary challenge in current research on *P. multocida* vaccines.

### 4.3. Attenuated Live Vaccines

Live attenuated vaccines constitute a category of immunological preparations containing live pathogens with reduced pathogenicity. These are traditionally divided into classically attenuated strains (derived from serial passage) and genetically engineered attenuated strains (with targeted gene deletions). The development of these vaccines generally employs techniques such as chemical mutagenesis or targeted deletion of virulence-associated genes. These methods diminish the virulence of the pathogen while preserving its immunogenic properties, enabling the vaccine strain to replicate to a limited degree within the host following administration. This controlled replication effectively simulates the natural infection process [47].

Live attenuated vaccines are capable of eliciting robust and durable immune responses within the host, encompassing humoral, cellular, and mucosal immunity, thereby conferring high levels of protective efficacy. Notably, naturally attenuated strains, such as the CU and P4675 strains, offer distinct advantages including prolonged immunity, extensive cross-protection, and reduced production costs. For instance, immunization with the CU strain vaccine—administered through drinking water, subcutaneous injection, or wing puncture—has demonstrated protection rates exceeding 95%, with efficacy markedly surpassing that of inactivated vaccines [47].

The protective efficacy and duration of live attenuated vaccines are influenced by multiple factors. Variables such as the route of administration, dosage, as well as the recipient’s age and immune status, can result in variability in the immune response elicited. Furthermore, attenuated strains exhibit sensitivity to specific antimicrobial agents [47]; consequently, the administration of these drugs should be avoided during the vaccination period to prevent interference with the replication of the vaccine strain and to maintain immunization effectiveness. Of particular concern is the potential for live attenuated vaccines to revert to a virulent form, regaining pathogenicity following repeated passages within the host. This safety issue restricts their broad application in certain animal populations. Hence, the deployment of live attenuated vaccines necessitates a comprehensive evaluation of factors including immunogenicity, safety, and appropriate usage conditions.

### 4.4. Recombinant Subunit Vaccines

PlpE, currently regarded as the most promising antigen candidate for subunit vaccines, represents the first recombinant protein demonstrated to confer cross-serotype protection [48]. Empirical evidence indicates that recombinant PlpE effectively protects both mice and chickens against infections caused by diverse serotypes [48]. Notably, its linear epitopes retain protective efficacy even in denatured states, and it is anticipated that multi-epitope constructs may induce broader immune responses compared to single-epitope formulations [49]. Additionally, the amino acid sequences of the PlpE protein exhibit a high degree of conservation among virulent *Pasteurella* strains isolated from these animals and humans, with sequence identity ranging from 90.8% to 100% [48].

Studies have shown that in recent years, the development of genomic vaccines against *P. multocida* has focused on various antigenic molecules with protective potential, among which the outer membrane lipoproteins VacJ and PlpE, as well as the outer membrane protein OmpH, are the key candidate targets in this field [50]. Empirical evidence indicates that recombinant OmpH (rOmpH) alone confers 83.33% protection in ducks, with its protective capacity increasing to 100% when administered in combination with recombinant PlpE (rPlpE) and recombinant VacJ (rVacJ) against homologous *P. multocida* challenge [50]. Furthermore, recent investigations employing dendritic cell-targeting peptides fused to the Salmonella FliCd flagellin protein and OmpH have markedly augmented protective efficacy across multiple serotypes [51].

Iron-regulated outer membrane proteins (IROMPs), such as Transferrin-binding protein A (TbpA) [52], represent critical virulence factors in pathogenic strains responsible for hemorrhagic septicemia (HS), while they are absent in non-HS serotypes. Vaccination with TbpA has been shown to elicit robust humoral and cellular immune responses, with enhanced protective efficacy observed when TbpA is co-expressed alongside the interleukin-2 (IL-2) gene [52,53]. Furthermore, back-passage of vaccine seed strains in the natural host significantly enhances their immunogenicity [54].

Several other outer membrane proteins (OMPs), including OmpA, VacJ, and PmSLP, have exhibited strong immunogenic properties and conferred effective protective immunity. Innovative fusion proteins, such as PlpEC-OmpH, have been shown to achieve protection rates ranging from 60% to 80% [55]. Research findings suggest that the simultaneous administration of multiple protective antigens elicits a broader and more robust immune response compared to the use of a single antigen, thereby offering a theoretical foundation for the development of multivalent subunit vaccines.

### 4.5. DNA Vaccines

DNA vaccines stimulate an immune response by encoding essential protective antigens, including OmpH. Gong et al. developed a naked DNA vaccine targeting the ptfA gene, which encodes a key protective antigen of avian *P. multocida* [32]. This DNA vaccine was subsequently encapsulated within chitosan, serving dual functions as both a carrier and an adjuvant, thereby successfully creating a chitosan nanoparticle DNA vaccine. The study optimized the vaccine formulation process and conducted a comprehensive characterization of encapsulation efficiency, morphology, and stability. These efforts established a foundation for assessing the immunogenicity of the candidate vaccine and contributed to advancing novel DNA vaccine strategies against avian *P. multocida* [32].

Previous findings have demonstrated that rats receiving either the DNA-based vaccine or the inactivated vaccine exhibited markedly elevated serum antibody titers. Furthermore, although the DNA vaccine conferred only partial immunity against challenge, its overall protective effect was found to be superior to that induced by the live-attenuated vaccine [33]. While DNA vaccines offer advantages such as straightforward preparation and favorable stability, their broader application is constrained by inherently low immunogenicity. The employment of chitosan nanoparticles as delivery systems for DNA vaccines can effectively shield plasmid DNA from nuclease degradation and enhance antigen presentation, attributable to their mucosal adhesive properties [32].

### 4.6. Recombinant Vector Vaccines

Recombinant vector vaccines are developed through genetic engineering techniques that enable the direct delivery of antigen-encoding genetic material into host cells, thereby eliciting robust cellular and humoral immune responses that often surpass those achieved by conventional inactivated or subunit vaccines [56]. Upon administration, the recombinant microorganism expresses the target antigen protein within the host, thereby activating the host’s immune system to generate specific antibody-mediated and cellular immune responses against the pathogen of interest [57]. For instance, the use of attenuated Salmonella strains (e.g., S. Choleraesuis rSC0016) as vectors to express the PlpE protein has been shown to induce robust mucosal, humoral, and cellular immunity. Oral immunization with this recombinant vaccine conferred an 80% protection rate against a lethal challenge in murine models, surpassing the 60% protection afforded by an inactivated vaccine [58].

In a similar approach, Apinda et al. utilized CRISPR/Cas9 gene-editing technology to insert the exogenous gene encoding OmpH, a protective antigen of *P. multocida*, into the genome of an attenuated duck enteritis virus (DEV) strain, successfully constructing two recombinant viral vector vaccines. These recombinant vaccines were able to stably express the OmpH protein during in vitro passaging without compromising viral replicative capacity. Immunization experiments demonstrated that ducklings vaccinated with these recombinant vector vaccines simultaneously induced specific antibody responses against both DEV and *P. multocida*, providing complete protection against both duck plague and fowl cholera. This study further confirmed that the DEV vector modified via CRISPR/Cas9 technology can serve as a multivalent recombinant vaccine platform for expressing bacterial antigens, offering an important technical strategy for the development of recombinant vector vaccines [57].

Recombinant vector vaccines hold potential not only for the prophylaxis of infectious diseases but also as therapeutic agents in immunotherapy, including cancer treatment. Compared to conventional vaccines, recombinant vector vaccines present several advantages: they typically require shorter development timelines and incur relatively lower production costs, while simultaneously eliciting more robust protective immune responses [56].

### 4.7. Outer Membrane Vesicle (OMV) Vaccines

Outer membrane vesicles (OMVs) are spherical, bilayered nanostructures ranging from 20 to 300 nm in diameter, which are secreted by Gram-negative bacteria during their growth phase. These vesicles comprise a diverse array of constituents, including lipopolysaccharides (LPS), outer membrane proteins, periplasmic proteins, and nucleic acids, and exhibit intrinsic immunostimulatory capabilities. OMVs present several advantages as vaccine platforms: the pathogen-associated molecular patterns (PAMPs) inherently present within OMVs can robustly activate the innate immune system, eliciting a potent immune response without the necessity for supplementary adjuvants. Additionally, OMVs can be delivered via mucosal routes, facilitating localized IgA production, and they pose no risk of reversion to a live pathogenic form.

**Figure 2:**
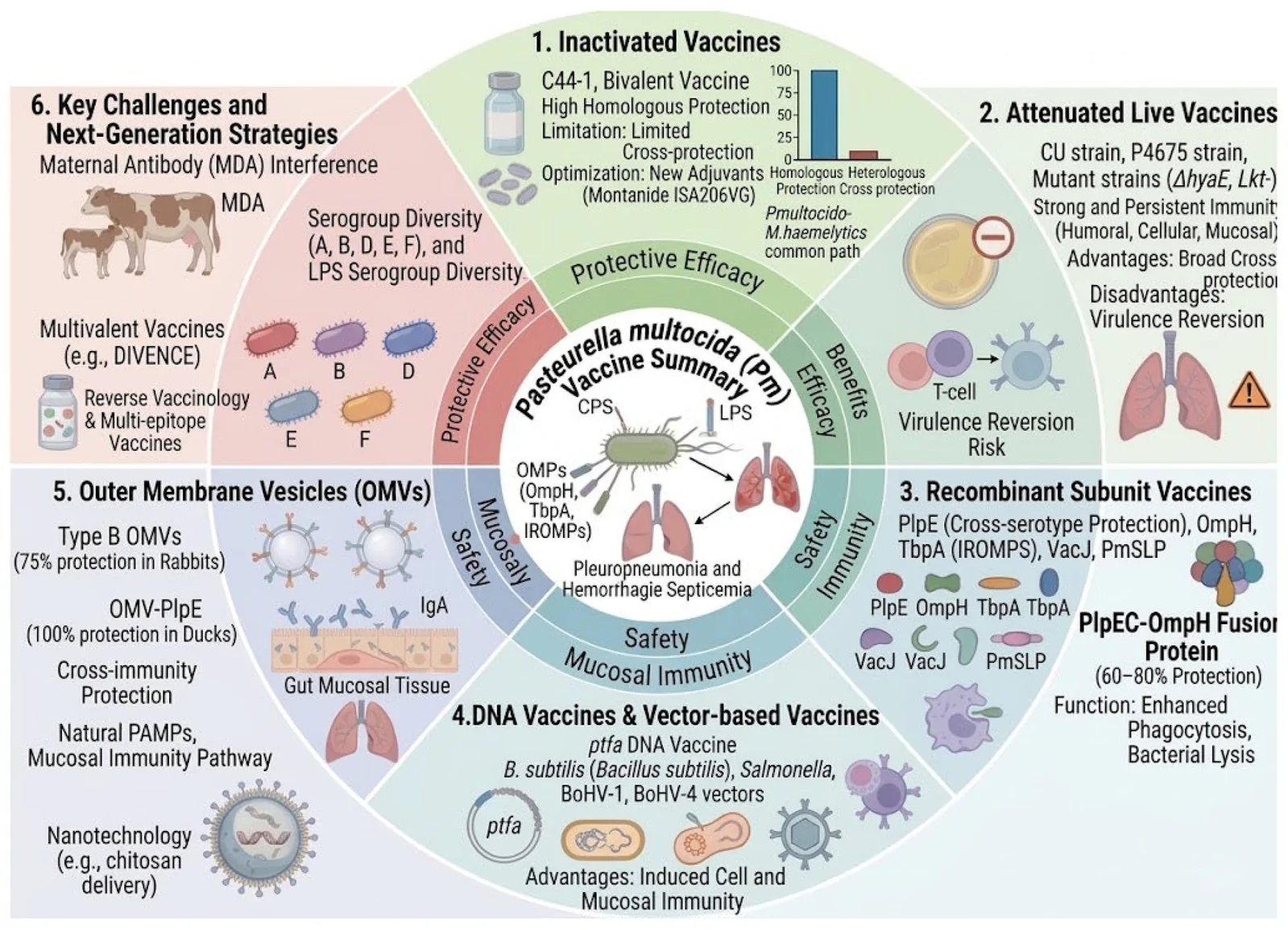
Vaccine development landscape for *P. multocida*.

Immunization of New Zealand white rabbits with outer membrane vesicles (OMVs) derived from *P. multocida* type B via intramuscular injection elicited serum antibody titers reaching up to 1:405,600 and conferred a 75% protection rate against challenge with a virulent strain by intramuscular challenge [59]. Similarly, immunization of ducks with *Escherichia coli*-derived OMVs containing PlpE (OMV-PlpE) induced robust specific antibody responses and significantly upregulated the expression of cytokines IL-2, IL-4, IL-10, and IFN-γ. This immunization provided complete (100%) protection against a 20-fold lethal dose 50 (LD50) challenge, while markedly reducing tissue damage and bacterial colonization [60]. OMVs are recognized as potent inducers of both humoral and mucosal immune responses. When delivered via the intranasal route, they have been shown to effectively reduce bacterial colonization within the nasopharynx. Moreover, OMVs derived from distinct bacterial species can confer cross-protective immunity against heterologous strains. A notable practical advantage is their remarkable thermal stability, which allows for prolonged storage at room temperature for several months without compromising immunogenicity—a feature that greatly facilitates vaccine distribution and field application (Figure 2).

Similarly, Table 2 provides a systematic comparison of *P. multocida* vaccine platforms, covering the full spectrum from traditional inactivated vaccines to novel OMV and recombinant vector-based vaccines, with corresponding literature support.

The figure summarizes the major vaccine platforms and key challenges for *P. multocida*. Inactivated vaccines (e.g., C44-1, bivalent vaccines) provide high homologous protection but limited cross-protection; optimization with novel adjuvants (e.g., Montanide ISA 206 VG) is underway. Attenuated live vaccines (e.g., CU strain, P4675 strain, Δ*hyaE*, Lkt^−^ mutants) induce strong and persistent humoral, cellular, and mucosal immunity with broad cross-protection but carry the risk of virulence reversion. Recombinant subunit vaccines include PlpE (cross-serotype protection), OmpH, TbpA (IROMPs), VacJ, and PmSLP. DNA vaccines and vector-based vaccines utilize platforms such as ptfA DNA vaccine, Bacillus subtilis, Salmonella, BoHV-1, and BoHV-4 vectors. Outer membrane vesicles (OMVs), including type B OMVs (75% protection in rabbits) and OMV-PlpE (100% protection in ducks), offer advantages such as natural PAMPs, mucosal immunity pathway activation, and cross-immunity protection. Key challenges include maternal antibody (MDA) interference and serotype diversity (capsular types A, B, D, E, F and LPS genotypes). Next-generation strategies feature multivalent vaccines (e.g., DIVENCE), nanotechnology (e.g., chitosan delivery), reverse vaccinology, and multi-epitope vaccines.

## 5. Combined Vaccines and Comprehensive Prevention and Control of BRD

### 5.1. Multivalent Combination Vaccines

Considering the multi-pathogenic etiology of bovine respiratory disease (BRD), the advancement of multivalent vaccines incorporating both viral and bacterial antigens represents a critical strategy. The recently introduced DIVENCE vaccine comprises a gE/tk double-deletion bovine herpesvirus type 1, an attenuated bovine respiratory syncytial virus, inactivated parainfluenza virus type 3, and recombinant proteins of bovine viral diarrhea virus types 1 and 2. This vaccine has demonstrated a significant reduction in BRD incidence (26.78% compared to 41.24%) and antibiotic usage (33.3% versus 57.4%) [61]. Field evaluations further reveal that the DIVENCE vaccine effectively diminishes both the occurrence and severity of BRD in commercial feedlot settings, lowers the requirement for antimicrobial interventions, and notably enhances carcass weight by 6.58 kg [61]. Additionally, an attenuated live vaccine containing the leukotoxin-mutant strain of *M. haemolytica* A1 and the Δ*hyaE*-mutant strain of *P. multocida* has been shown to confer cross-protection against *M. haemolytica* A6, resulting in reductions in clinical symptoms, pulmonary lesion scores, and mortality rates [62].

The recombinant bovine herpesvirus (BoHV) vector platform represents one of the cutting-edge strategies for addressing the challenges in developing multivalent vaccines against bovine respiratory disease complex (BRD). The core advantage of this strategy lies in exploiting the large genomic capacity and high-efficiency expression characteristics of herpesviruses, enabling them to carry and deliver protective antigens derived from other key pathogens [63]. Currently, candidate vaccines based on BoHV-1 and BoHV-4 vectors have demonstrated potential in both experimental and target animal models, including the successful induction of specific neutralizing antibodies against bovine viral diarrhea virus (BVDV) as well as protection against homologous virus challenge [64]. Collectively, these preliminary explorations suggest that this vector system holds broad application prospects in the development of multivalent vaccines targeting the complex etiologies of BRD [63].

Regarding the field application of BRD multivalent combination vaccines, Table 3 compares different multivalent formulations in terms of pathogens covered, key components, immune efficacy, field results, route of administration, and known limitations, enabling readers to quickly assess the practical value of each vaccine.

### 5.2. Field Application Results

Crouch et al. (2012) demonstrated that an attenuated live vaccine containing the leukotoxin-mutant strain of *M. haemolytica* A1 and the ΔhyaE-mutant strain of *P. multocida* conferred cross-protection against *M. haemolytica* A6, resulting in reductions in clinical symptoms, pulmonary lesion scores, and mortality rates [62]. This cross-protective capacity is particularly relevant given the recent emergence of serotype A6 as a predominant field strain not covered by many traditional commercial vaccines.

Earlier field studies from the 1990s established foundational principles for BRD vaccination. Harland et al. (1992) found that the efficacy of a recombinant *M. haemolytica* vaccine could be influenced by concurrent administration of subunit or modified-live bovine herpesvirus-1 vaccines, suggesting potential interference effects [67]. Van Donkersgoed et al. (1994) evaluated a combined *M. haemolytica* and *H. somnus* bacterin administered alongside a modified-live bovine respiratory syncytial virus (BRSV) vaccine, finding that this combination did not significantly reduce the incidence of enzootic pneumonia [68]. Five field trials investigating BRSV vaccines confirmed their efficacy in reducing medical treatment costs, with benefits closely linked to vaccination timing and herd health status [69]. Additionally, the prophylactic use of parenteral antibiotics for early respiratory disease exhibited limited and inconsistent effectiveness, underscoring the critical role of vaccination in BRD control [70].

While these early studies established important principles—such as the multifactorial nature of BRD protection, the impact of vaccine interference, and the influence of management factors—they were conducted prior to the taxonomic reclassification of Pasteurella/Mannheimia and before many contemporary commercial vaccine formulations were developed. As such, their specific efficacy data should be interpreted with appropriate caution when applied to current field conditions.

Zhou et al. (2025) conducted an epidemiological investigation in northern China, identifying viral-bacterial co-infection as the primary etiological factor underlying BRD, providing a theoretical basis for multivalent combination vaccines [71]. O’Donoghue et al. (2025) comprehensively reviewed BRD epidemiology, diagnostic challenges, and vaccination strategies, emphasizing that vaccine effectiveness depends on multiple interacting factors [1].

Collectively, these findings demonstrate that vaccine efficacy is modulated by management practices, stress, maternal antibodies, vaccine compatibility, and production environment. However, given that much of the historical field data predates taxonomic reclassification and current commercial vaccines, these earlier results should be interpreted as establishing enduring principles of BRD immunology rather than providing direct measures of contemporary vaccine performance. For current practice, recent trials evaluating commercially available products in modern production settings—such as the DIVENCE vaccine trial [61] and the Japanese Wagyu calf study [7]—offer more directly applicable evidence. The integration of historical knowledge with emerging data provides the most robust foundation for guiding future research and prevention strategies.

## 6. Key Challenges in Vaccine Development

### 6.1. Interference from Maternal Antibodies

Maternal antibodies (MDAs) present in calves can substantially impede the effectiveness of vaccines. Research has demonstrated that maternal antibodies against bovine respiratory syncytial virus (BRSV) suppress the humoral immune response elicited by vaccination in calves, although they do not prevent the activation of the cellular immune system [72,73]. The levels of MDAs exhibit considerable variability among dairy calves, with neutralizing antibody titers ranging from less than 1:16 to greater than 1:512. Elevated MDA titers are correlated with a decreased incidence of bovine respiratory disease (BRD), yet they also markedly diminish vaccine efficacy [73]. In a study involving 102 beef calves, Fulton et al. (2004) [74] found that maternally derived antibodies significantly interfered with the immunogenicity of vaccines against *M. haemolytica* and *P. multocida*. The study also determined the half-lives of maternally derived antibodies against multiple pathogens in calves (e.g., approximately 23 days for BVDV and 21 days for BHV-1), with bacterial antigen-specific maternal antibodies likewise showing a declining trend over time. Although calves with maternal antibodies were still able to mount a certain level of active immune response following vaccination, the seroconversion rate (defined as a four-fold or greater increase in antibody titers) remained generally low. These findings underscore the urgent need to develop novel vaccine platforms capable of circumventing maternal antibody interference or to optimize existing immunization regimens.

Mucosal immunization has been identified as a potentially effective strategy to mitigate the adverse effects of maternal antibodies on the immunization of calves [75]. Epidemiological studies have demonstrated a significant correlation between the incidence of bovine respiratory disease in calves and factors such as the status of maternally derived antibodies, farm environmental management practices, and the duration of exposure to pathogenic microorganisms [76]. Furthermore, immunological data indicate that cellular immune responses can be effectively elicited despite the presence of maternal antibodies, offering novel insights for the refinement of existing vaccination protocols in calves [75].

### 6.2. Adjuvants and Delivery Systems

Adjuvants play a crucial role in augmenting the immunogenicity of vaccines. In the context of veterinary vaccines, the adjuvants most frequently employed can be categorized as follows: oil-in-water (O/W) formulations, exemplified by Montanide™ ISA 206 VG and Freund’s adjuvant [18], which are effective in eliciting durable immune responses but may induce localized adverse reactions; aluminum-based adjuvants, noted for their favorable safety profiles albeit with comparatively modest immunostimulatory capacity; and microbial-derived adjuvants, including Bacillus subtilis spores and cholera toxin subunit B (CTB), which are proficient in stimulating mucosal immunity. Additionally, nanocarrier systems, such as chitosan nanoparticles, have demonstrated the ability to enhance immunogenicity through improved antigen presentation [32]. Furthermore, the optimization of immunization routes and dosing regimens exerts a significant influence on vaccine efficacy, as varying vaccination protocols can result in differential serological responses [68]. Consequently, the development of an ideal adjuvant or delivery system necessitates a careful balance between maximizing immunogenic potential and maintaining safety, a consideration that is especially critical when designing vaccines for young animals or those affected by maternal antibody interference.

The optimization of mucosal adjuvants and delivery systems is essential for eliciting localized immunity within the respiratory tract. Given that bovine respiratory disease (BRD) pathogens predominantly enter through the respiratory mucosa, vaccines capable of inducing mucosal IgA antibodies are likely to be more efficacious than those that solely stimulate serum IgG responses. Intranasal administration of outer membrane vesicles (OMVs) [77], plant-derived oral vaccines [27], and Bacillus spore-based vaccines [29,30] represent promising approaches for mucosal immunization. These methodologies not only promote local IgA production but also confer protective immunity at distal mucosal sites via the interconnected mucosal immune network.

### 6.3. Cross-Protection and Serotype Coverage

The limited cross-protection observed among different serotypes of *M. haemolytica* (specifically A1, A2, and A6) constitutes a significant obstacle in vaccine development. Notably, the leukotoxin A (LktA) protein demonstrates substantial genetic variability, with previous studies reporting nucleotide divergence ranging from 1.4% to 15.7% among different lktA allelic variants across serotypes [78], and the heterogeneity of outer membrane proteins (OMPs) further constrains the efficacy of cross-protective immunity. Similarly, *P. multocida* presents a wide array of capsular serotypes and lipopolysaccharide (LPS) variants, complicating the ability of monovalent vaccines to confer broad-spectrum protection. Consequently, future vaccine research and development efforts are directed towards the formulation of multivalent vaccines—such as trivalent vaccines targeting *M. haemolytica*—or the design of subunit vaccines that focus on conserved antigenic components, including PlpE and OmpH.

Outer membrane vesicle (OMV) vaccines exhibit distinct advantages regarding cross-protective efficacy [77]. Research has indicated that immunization with OMVs derived from *M. haemolytica* can elicit cross-reactive antibodies targeting *P. multocida*, and conversely, immunization with *P. multocida* OMVs induces antibodies reactive to *M. haemolytica* [77]. These findings suggest that conserved antigenic components within OMVs may confer broad-spectrum immunological protection. The cross-protective capacity of OMV-based vaccines holds significant potential for the development of comprehensive vaccines targeting multiple pathogens associated with bovine respiratory disease (BRD).

### 6.4. Correlates of Protection and Regulatory Considerations

The establishment of correlates of protection is a critical step in rational vaccine design and regulatory approval. For *M. haemolytica* and *P. multocida* vaccines, no universally accepted immunological correlates of protection have been established, which constitutes a major obstacle to vaccine development.

Regarding serological correlates, anti-LktA neutralizing antibody titers are considered to correlate with protective efficacy, as high-titer antibodies can neutralize the cytotoxic effects of leukotoxin [15]. However, antibody titers alone do not always correlate proportionally with protective efficacy; antibody subclasses (e.g., IgG1/IgG2 ratio) may have greater predictive value. For *P. multocida*, both anti-capsular polysaccharide and anti-outer membrane protein antibodies correlate with protection, but cross-reactivity between serotypes is limited [5]. Regarding cellular immunity, Th1-type responses (marked by IFN-γ) play an important role in combating infection [17]. Mucosal IgA also plays a critical role in preventing pathogen colonization.

From a regulatory perspective, the absence of recognized correlates of protection makes it difficult to compare the efficacy of different vaccine candidates and increases uncertainty in vaccine approval. Currently, regulatory agencies primarily rely on challenge-protection studies to evaluate vaccine efficacy, but the lack of standardization in challenge models across different laboratories further complicates the evaluation process. Future research should focus on establishing standardized assays to identify reliable correlates of protection.

### 6.5. Challenge Models and Protection Data Interpretation

The challenge models used in the literature are diverse and lack standardization, which poses challenges to direct comparisons of protection rates across studies. Commonly used models include murine, rabbit, goat, and bovine models. The murine model is low-cost and easy to handle, but mice differ from cattle in susceptibility to *M. haemolytica* leukotoxin, as well as in respiratory tract anatomy and immune system architecture; therefore, results cannot be directly extrapolated [15,22]. The rabbit model is commonly used in hemorrhagic septicemia vaccine research [41], but susceptibility to different *P. multocida* serotypes varies. The goat model is used as an intermediate host in some studies, but its similarity to cattle is not fully understood. The bovine model most closely resembles field conditions, but its high cost and operational complexity limit its application in high-throughput screening.

Furthermore, considerable variation exists across studies in challenge doses, challenge routes (intratracheal, intranasal, aerosol inhalation, etc.), challenge strains (homologous/heterologous), and protective efficacy evaluation metrics (mortality, clinical scores, lung lesion scores, bacterial burden, etc.), making direct comparison of protection rates across different studies extremely difficult. Readers should carefully consider these differences when interpreting protection data from different experimental systems.

### 6.6. Safety Considerations: LPS Reactogenicity and Injection Site Reactions

For vaccines derived from Gram-negative bacteria, lipopolysaccharide (LPS) endotoxin-associated reactogenicity is a safety concern that cannot be overlooked. Residual LPS in vaccines can activate the innate immune system through TLR4, inducing the release of pro-inflammatory cytokines. Moderate amounts of LPS can exert a natural adjuvant effect, but excessive LPS may lead to fever, anorexia, acute-phase responses, and even endotoxic shock [19]. To mitigate LPS-associated adverse effects, researchers have explored multiple strategies, including chemical or physical removal of LPS, genetic modification of LPS biosynthesis genes, and regulation of the lipid A structure through OMV engineering.

Furthermore, oil-based adjuvants (e.g., Montanide™ ISA 206 VG, Freund’s complete adjuvant) can induce granuloma formation, sterile abscesses, or local tissue necrosis at the injection site, which not only affect animal welfare but may also cause carcass damage, impacting meat quality [18]. Aqueous and aluminum-based adjuvants cause milder local reactions but often have weaker immunopotentiating effects. Therefore, a careful balance between adjuvant potency and local reactogenicity is required in vaccine formulation design. The development of novel nano-adjuvants and mucosal delivery systems offers new possibilities for reducing injection site reactions while maintaining immunological efficacy.

### 6.7. Limitations of Species Extrapolation

Some studies cited in this review used non-bovine animal models (including mice, chickens, ducks, goats, and sheep) to evaluate vaccine efficacy. While these models have important value in early screening, caution is required when extrapolating their results to cattle.

First, there are significant differences in the immune systems of different species; mice and cattle differ in TLR expression profiles, cytokine networks, and antibody subclass distribution. Second, the leukotoxin of *M. haemolytica* exhibits much higher cytotoxic activity against bovine leukocytes than against murine leukocytes, which may lead murine models to underestimate the protective value of certain antigens [15,22]. Similarly, the pathogenicity of different *P. multocida* serotypes varies across host species [5]. Third, differences in respiratory tract anatomy make it difficult for small animal models to fully simulate the pathogenesis of bovine BRD.

Therefore, any candidate vaccine identified in small animal models must be validated through bovine challenge experiments before field deployment. This review has clearly specified the species, immunization route, and challenge model used for each cited study, enabling readers to fully consider the impact of species differences when interpreting the data.

### 6.8. Regional Epidemiological Disparities and Their Implications for Vaccine Selection

While the preceding sections have addressed specific epidemiological phenomena such as AMR trends and serotype shifts, it is important to recognize that the prevalence of *M. haemolytica* and *P. multocida* serotypes, as well as their antimicrobial resistance profiles, exhibits substantial geographic variation. These regional disparities have direct implications for vaccine selection and immunization protocols, yet they are often overlooked in broad-scope reviews.

In North America, A1 remains the most common *M. haemolytica* serotype in feedlot cattle, but A6 is increasingly reported [16,34]. Vaccines targeting A1 offer reasonable protection, but the rise of A6 suggests reformulation may be needed. In contrast, European surveillance data indicate a more heterogeneous serotype distribution, with A2 and A6 isolates comprising a larger share of clinical cases in some countries [11].

In China, surveillance data from different regions and detection methods have yielded varying prevalence rates, reflecting the diversity of cattle production systems and sampling strategies across the country. A comprehensive investigation in northern China covering 5052 samples from six provinces identified *P. multocida* and *M. haemolytica* as the most prevalent bacterial pathogens, with BRD prevalence in the Inner Mongolia cohort being 2.3-fold higher during winter than in summer, and identified calves under 3 months of age and beef calves over 3 months as high-risk groups [71]. In Northeast China (160 lung samples from dead cattle, 2016–2021), *P. multocida* and *M. haemolytica* were detected at rates of 8.35% and 2.44%, respectively, with *P. multocida* serotype A accounting for 91.67% (33/36) of isolates [79]. In Guizhou Province, a large-scale surveillance conducted from 2022 to 2025 reported a *P. multocida* detection rate of 20.76% [80]. A survey of sheep-derived *M. haemolytica* in northwestern China identified A1, A2, and A6 [81]. Genomic analysis of *P. multocida* strains in China has identified the dominant clone A:L1:ST129 in poultry and characterized its antimicrobial resistance gene repertoire [82]. Additionally, in yak populations, seroprevalence of *Pasteurella* infections was reported at 15.71% in Tibet [83].

For *P. multocida*, the regional picture is equally complex. Hemorrhagic septicemia caused by serogroup B remains a major concern in South and Southeast Asia, while serogroup A is more commonly associated with BRD in North America and Europe [5]. Within serogroup A, the distribution of LPS genotypes (L1–L8) also varies geographically, and vaccines effective against one genotype may offer little or no cross-protection against another [46]. This genotypic diversity complicates the selection of appropriate commercial vaccines for different production regions.

Comparison across regions shows commonalities and differences (Table 4). While the emergence of *M. haemolytica* A6 appears to be a shared trend across North America and China, the high prevalence of *P. multocida* type A in Chinese BRD cases contrasts with the dominance of type B in South Asian hemorrhagic septicemia outbreaks. Such regional variability underscores that effective BRD control requires locally adapted vaccine formulations and immunization schedules.

From a practical standpoint, these regional disparities underscore three key points. First, vaccine selection should ideally be guided by local or regional surveillance data rather than relying on assumptions based on studies from other continents. Second, the expanding epidemiological evidence from China—including the substantial prevalence of A6, the predominance of *P. multocida* capsular type A (91.67% in Northeast China), and high rates of multidrug resistance observed in some studies [82]—provides a strong rationale for developing region-specific multivalent vaccines targeting locally prevalent serotypes and for implementing resistance surveillance programs. Third, interpretation of vaccine efficacy data from field trials must consider regional serotype and resistance contexts, as results from one country may not be directly generalizable to another.

## 7. Next-Generation Vaccine Strategies

The reverse vaccinology workflow involves whole-genome sequencing, pan-genome analysis, and homologous sequence alignment to identify conserved surface proteins [84]. For multi-epitope design, bioinformatic tools predict B- and T-cell epitopes, which are then concatenated with flexible linkers [35,36]. Leveraging these genomic insights, multi-epitope vaccines have been developed based on bioinformatic predictions of conserved immunodominant epitopes derived from proteins including PlpE and OmpH. Empirical evidence suggests that multi-epitope formulations may induce broader protective immunity compared to single-epitope vaccines; however, the processes of epitope selection and arrangement require further refinement.

As discussed in Section 4.2, mucosal immunization offers a key strategy to circumvent maternal antibody interference and induce local IgA responses. In a complementary approach, precision vaccinology utilizes epidemiological data from field studies to tailor vaccines to the serotypes prevalent in specific geographic regions. For instance, Crouch et al. [62] demonstrated that their *M. haemolytica* A1–*P. multocida* combination vaccine conferred cross-protection against serotype A6—a serotype now recognized as one of the most prevalent clinical isolates from cattle. This finding is particularly relevant given that many current commercial vaccines do not claim or provide cross-protection against A6. Such serotype mismatches highlight the urgent need to incorporate A6 and other emerging predominant serotypes into next-generation vaccine formulations.

Beyond conventional vaccination approaches, immune modulation and innate immune training represent innovative avenues for BRD management. Research has demonstrated that immune modulators such as β-glucan can potentiate host resistance to subsequent infections by priming the innate immune system [85]. This approach does not target specific pathogens but rather amplifies nonspecific host immunity, serving as a complementary strategy alongside vaccination. Additionally, chimeric protein vaccines, exemplified by the PlpE-LKT fusion protein, have exhibited robust immunogenicity and protective efficacy in cattle, offering valuable insights for the development of next-generation vaccines [86].

## 8. Conclusions

Considerable advancements have been achieved in the development of vaccines targeting *M. haemolytica* and *P. multocida*. The evolution from conventional inactivated whole-cell vaccines to contemporary approaches—including recombinant subunit vaccines, DNA vaccines, vector-based vaccines, and outer membrane vesicle vaccines—has led to continuous improvements in both vaccine safety and efficacy. Future vaccine development efforts should prioritize: (1) addressing the challenge of maternal antibody interference and formulating effective immunization protocols tailored for neonatal animals; (2) enhancing cross-protective capabilities to encompass predominant circulating serotypes, with particular attention to the recently identified serotype A6; (3) optimizing adjuvants and delivery mechanisms, especially via mucosal immunization routes, to elicit robust localized immune responses; and (4) creating multivalent combination vaccines to facilitate comprehensive prevention and control of bovine respiratory disease (BRD). By incorporating advanced methodologies such as reverse vaccinology, structural biology, nanotechnology, and immunoinformatics, it is anticipated that next-generation vaccines with improved safety profiles, heightened efficacy, and broader protective spectra will be developed, thereby supporting the sustainable health and productivity of the cattle industry.

## Figures and Tables

**Table 1:** Summary of *M. haemolytica* vaccine platforms.

Vaccine Platform	Key Antigens	Adjuvant/Delivery System	Immune Response Type	Protective Efficacy (Species/Model)	Advantages	Limitations	Representative References
Inactivated whole-cell	Whole-cell antigens, LktA, OMPs	Oil-based/aluminum adjuvants	Primarily humoral (IgG)	Homologous protection (cattle)	High safety, no reversion risk	Limited cross-protection, multiple doses required	[14,16,17]
Recombinant subunit (cLktA)	LktA C-terminal domain	Freund’s/Montanide	Neutralizing antibodies	80–100% (mice/goats, intramuscular)	Well-defined, high safety	Single-antigen limitations	[22]
Recombinant subunit (rPlpE)	PlpE OMP	Oil-based adjuvants	Complement-mediated bactericidal antibodies	Enhanced protection (cattle)	Cross-serotype expression	Degradation during purification	[23,24]
Recombinant subunit (rSsa1)	Ssa1 OMP	Oil-based adjuvants	Antibody response	Immunogenic in calves	Serotype-specific antigen	Cross-protection not fully established	[26]
Plant-based oral vaccine	LktA + PlpE + CTB	*Nicotiana benthamiana* expression	Mucosal IgA + serum IgG	Immunogenic in mice	Low cost, scalable, no cold-chain	Antigen yield and stability	[27,28]
Bacillus spore-based vaccine	MhCP (LktA + PlpE epitopes)	B. subtilis spores (self-adjuvanting)	Secretory IgA (mucosal)	Enhanced bactericidal efficacy (mice)	Thermostable, no cold-chain, mucosal	Field data limited	[29,30,31]
Nanoparticle delivery (prospective)	DNA vaccines (e.g., ptfA)	Chitosan nanoparticles	Humoral + cellular + mucosal	Promising in related species *(P. multocida* models)	Mucoadhesive, protects plasmid DNA	*M. haemolytica*-specific data limited	[32,33]
Trivalent vaccine (A1 + A2 + A6)	Whole cells + rLktA + rSSA-1	Montanide™ ISA 206 VG	Neutralizing antibodies	100% protection (cattle)	Broad serotype coverage	Complex formulation	[16]
Multi-epitope vaccine (prospective)	Concatenated B/T-cell epitopes	Immunoinformatics design	Precise immune activation	To be validated	Broad-spectrum, no serotype limitation	Still in development	[35,36]

**Table 2:** Summary of *P. multocida* vaccine platforms.

Vaccine Platform	Key Antigens	Adjuvant/Delivery System	Immune Response Type	Protective Efficacy (Species/Model)	Advantages	Limitations	Representative References
Inactivated whole-cell (OAV)	Whole-cell antigens	Oil-based adjuvants	Primarily humoral	Homologous 100%, heterologous 0% (mice)	High safety, well-established	Poor cross-protection, short-lived immunity	[41,45,46]
Live attenuated	Whole-cell live antigens (CU, P4675)	None required	Humoral + cellular + mucosal	>95% (avian)	Durable immunity, broad cross-protection	Virulence reversion risk	[47]
Recombinant subunit (rPlpE)	PlpE	Oil-based adjuvants	Cross-protective antibodies	Effective in mice and chickens	Cross-serotype protection	Single-antigen immunogenicity limited	[48,49]
Recombinant subunit (rOmpH)	OmpH	FliCd + DC-targeting peptide	Enhanced Th1/Th2	83.3–100% (ducks)	Targeted delivery, combination potential	Optimization needed	[50,51]
Recombinant subunit (rTbpA)	TbpA (IROMP)	IL-2 co-expression	Humoral + cellular	Enhanced (bovine/murine)	HS-specific, strong immunogenicity	Absent in non-HS serotypes	[52,53]
Recombinant fusion (PlpEC-OmpH)	PlpE + OmpH fusion	Oil-based adjuvants	Broad antibody response	60–80% (mice)	Multivalent single construct	Combination optimization needed	[55]
DNA vaccine	ptfA, OmpH	Chitosan nanoparticles	Humoral + cellular	Superior to live-attenuated (rats)	Stable, mucosal delivery possible	Immunogenicity needs improvement	[32,33]
Recombinant vector (Salmonella)	PlpE	Attenuated Salmonella	Mucosal + humoral + cellular	80% (mice)	Mucosal immunity, multivalent potential	Vector interference effects	[58]
Recombinant vector (DEV)	OmpH	CRISPR/Cas9-edited DEV	Humoral + cellular	Complete protection (ducks)	Bivalent (DEV + *P. multocida*)	Host-specific vector	[57]
OMV vaccine (type B)	LPS, OMPs, natural PAMPs	Self-adjuvanting	Humoral + mucosal	75% (rabbits, intramuscular)	Natural adjuvant, no reversion risk	Endotoxin safety concerns	[59]
OMV-PlpE (chimeric)	PlpE displayed on OMVs	Self-adjuvanting	Robust antibody + cytokines	100% (ducks)	Enhanced immunogenicity	Endotoxin safety concerns	[60]
PmSLP3-based subunit	PmSLP3 surface lipoprotein	Montanide ISA 61	Sustained IgG	Full protection against B/E (cattle)	Long-lasting (up to 3 years)	Requires two doses	[42]

**Table 3:** Comparison of BRD multivalent combination vaccines.

Vaccine Name/Formulation	Pathogens Covered	Key Components	Immune Efficacy	Field Results	Route of Administration	Known Limitations	Representative References
DIVENCE	BHV-1, BRSV, PI-3, BVDV-1/2	gE/tk-deleted BHV-1 + attenuated BRSV + inactivated PI-3 + BVDV recombinant proteins	BRD incidence reduced (26.78% vs. 41.24%)	Reduced antibiotic use; carcass weight +6.58 kg	Intramuscular	Viral only (no bacterial components); requires reconstitution	[61]
Trivalent bacterial vaccine	*P. multocida*, *M. haemolytica*, *H. somni*	Inactivated whole cells	BRD incidence 25.9% vs. 70.9% (OR = 0.143) in Japanese Wagyu calves	Significantly reduced BRD risk	Subcutaneous	Limited cross-serotype coverage (A1 only)	[7]
Attenuated bivalent vaccine	*M. haemolytica* A1 Lkt^−^ + *P. multocida* ΔhyaE	Lkt mutant + Δ*hyaE* mutant strains	Cross-protection against *M. haemolytica* A6	Reduced clinical scores, lung lesions, mortality	Intramuscular	Live vaccine; reversion risk	[62]
Combined vaccine (inactivated + rLkt)	*P. multocida* A + *M. haemolytica* A6 + rLkt	Inactivated cells + recombinant leukotoxin	100% (*P. multocida*); 80% (*M. haemolytica*) in mice	Markedly reduced pulmonary lesions	Intramuscular (murine)	Murine model only; field data pending	[65]
Bovilis MH + IBR	*M. haemolytica* + IBR	Inactivated cells + IBR vaccine	BRD risk ratio 0.47 in finishing cattle	Reduced growth rate in backgrounding phase	Intramuscular	Growth suppression in some phases	[66]

**Table 4:** Regional distribution of *M. haemolytica* and *P. multocida* serotypes in cattle.

Region	*M. haemolytica* Serotypes	*P. multocida* Types	Key Epidemiological Features	Vaccine Implications
North America	A1 (dominant), A6 (increasing) [16,34]	Capsular A	Feedlot cattle; A6 emergence	Current A1 vaccines need A6 coverage
Europe	Heterogeneous (A1, A2, A6) [11]	Capsular A	Variable distribution across countries	Broad-spectrum coverage required
China	Diseased cattle: A1 and A6; healthy cattle: A2 [79]; Sheep isolates: A1, A2, A6 [81]	Capsular A dominant (91.67% in NE China [79]); Avian: A:L1:ST129 dominant [82]; 20.76% detection in Guizhou [80]	Winter peak (2.3-fold higher vs. summer) [71]; calves < 3 mo and beef calves > 3 mo are high-risk groups [71]	A6-targeting vaccines; region-specific multivalent formulations; cross-host surveillance needed
South/Southeast Asia	Limited data	Capsular B [5]	Hemorrhagic septicemia major concern	HS-specific type B vaccines

## Data Availability

All data for this study are available within the article.
